# Diabetes Protects from Prostate Cancer by Downregulating Androgen Receptor: New Insights from LNCaP Cells and PAC120 Mouse Model

**DOI:** 10.1371/journal.pone.0074179

**Published:** 2013-09-10

**Authors:** Anna Barbosa-Desongles, Cristina Hernández, Ines De Torres, Francina Munell, Marie-France Poupon, Rafael Simó, David M. Selva

**Affiliations:** 1 Diabetes and Metabolism Research Unit, Vall Hebron Institut de Recerca (VHIR). Universitat Autònoma de Barcelona and Centro de Investigación Biomédica en Red de Diabetes y Enfermedades Metabólicas Asociadas (CIBERDEM, ISCIII), Barcelona, Spain; 2 Pathology Department, Hospital Vall d’Hebron, Universitat Autònoma de Barcelona, Barcelona, Spain; 3 Pediatric Neurology Department, Vall Hebron Institut de Recerca (VHIR). Universitat Autònoma de Barcelona, Barcelona, Spain; 4 Département D′Oncologie Médicale, Institut Curie, Paris, France; Queensland University of Technology, Australia

## Abstract

Type 2 diabetes has been associated with decreased risk of prostate cancer in observational studies, and this inverse association has been recently confirmed in several large cohort studies. However the mechanisms involved in this protective effect remain to be elucidated. The aim of the present study was to explore whether different features of type 2 diabetes (hyperinsulinemia, hyperglycemia and tumor necrosis factor alpha [TNF-α]) protect against the development of prostate cancer. For this purpose LNCaP cells were used for *in vitro* experiments and nude mice in which PAC120 (hormone-dependent human prostate cancer) xenografts had been implanted were used for *in vivo* examinations. We provide evidence that increasing glucose concentrations downregulate androgen receptor (AR) mRNA and protein levels through NF-κB activation in LNCaP cells. Moreover, there was a synergic effect of glucose and TNFα in downregulating the AR in LNCaP cells. By contrast, insulin had no effect on AR regulation. *In vivo* experiments showed that streptozotocin-induced diabetes (STZ-DM) produces tumor growth retardation and a significant reduction in AR expression in PAC120 prostate cancer mice. In conclusion, our results suggest that hyperglycemia and TNF-α play an important role in protecting against prostate cancer by reducing androgen receptor levels via NF-κB.

## Introduction

Type 2 diabetes (T2D) and prostate cancer (PCa) are two major, growing health problems that affect millions of men worldwide. PCa is an androgen-dependent malignancy which constitutes the most common solid organ cancer in men in the US, Canada and Australia, and the second most common cancer in men globally [1].

T2D has been recognized as a key factor contributing to the development of solid organ malignancies including liver, pancreas, colorectal, breast, endometrial, uterine, and bladder [2], [3] However, several large cohort studies have demonstrated a significant decreased risk of PCa in T2D [4]–[7]. In addition, it has been observed that the magnitude of this inverse association is higher with increasing duration of diabetes [4], [8]. Although long-standing diabetes protects against PCa development, there is evidence that diabetic men may have a worse outcome because they have hystologically more aggressive PCa in comparison with non-diabetic patients [9]–[11]. One of the reasons for this feature could be the lower serum levels of prostate-specific antigen (PSA) that type 2 diabetic patients present in comparison with nondiabetic subjects [5], [12], [13]. Since PSA is the current screening method for PCa, lower PSA levels that exist in diabetic patients could lead to diagnosis delay, thus increasing the incidence of high-grade/advanced PCa. However, it should be noted that the protective effect of diabetes on PCa is not simply a consequence of detection bias from delayed diagnosis due to lower PSA levels [5], [14], [15].

The precise mechanisms involved in the protective effect of T2D on PCa development remain to be elucidated. It has been reported that individuals with increased genetic susceptibility to T2D have a decreased risk of PCa [16]–[18]. In this regard it has been shown that the same variation in the HNF1B (also known as TCF2) gene, which is associated with increased PCa risk, confers protection against T2D [19]. Other explanations proposed have included the progressive development of beta cell exhaustion with insulin depletion and the association of diabetes with lower testosterone and insulin growth factor 1 (IGF1) levels [15]. In addition, it should be noted that low-degree inflammation is a significant component of T2D and that higher serum levels of tumor necrosis factor alpha (TNF-α) in comparison with nondiabetic controls have been reported [20]. Recently we provided evidence that TNFα may play an essential role in downregulating SHBG and serum levels of testosterone [21], [22]. Therefore, a cross-talk between inflammation and androgen levels could be a underlying mechanism accounting for the low risk of PCa observed in T2D. Furthermore, it has recently been shown that several inflammation-related genes are associated with serum androgen levels in men [23].

Since PCa is androgen-dependent, the first aim of the present study was to examine whether insulin or glucose levels had any effect on the androgen receptor (AR) in PCa LNCaP cells (an androgen-sensitive immortalized PCa cancer cell line) [24]. In addition, given that TNF-α is upregulated in T2D and a negative regulation of AR expression by TNF-α has been previously reported [25], we wanted to explore whether TNF-α had any effect on AR regulation in our *in vitro* system. Moreover, since hyperglycemia is able to activate NF-κB in endothelial cells, pericytes and vascular smooth muscle cells [26]–[30], and NF-κB activation might be involved in AR downregulation [25], [31], we examined whether hyperglycemia could reduce AR levels through NF-κB activation in LNCaP cells. Finally, a PAC120 prostate cancer mouse model [32] treated with streptozotocin (STZ) was used to evaluate *in vivo* the effects of hyperglycemia on AR regulation and tumor growth.

## Materials and Methods

### Cell Culture

The human prostate cancer cell lines LNCaP were obtained from the American Type Culture Collection (Rockville, MD, US) and were plated in 75 cm2 flasks and cultured in RPMI 1640 (PAA Laboratories, Pasching, Austria) containing 10% FBS, 1% Penicillin/Streptomycin, 1% Hepes and different insulin concentrations (20, 100 or 200 µIU/ml) or glucose concentration (5, 10 or 30 mM of D-glucose and 5 or 30 mM of L-glucose) for 4 days. LNCaP cells were also treated with increasing amounts of D-glucose (5 mM, 10 mM and 30 mM) in the presence or absence of TNFα (50 ng/ml) or QNZ (Enzo Life Sciences International, Inc. PA, USA). Cells were maintained in a humidified incubator with 5% CO_2_ at 37°C. All *in vitro* experiments were done at least in two independent experiments by triplicate.

### Animal Model

For *in vivo* studies a PAC120 PCa mouse model was used. De Pinieux *et al* [33] created this model of human PCa using tissue obtained by transurethral resection of a locally recurrent PCa. This PCa tissue was established as the transplantable xenograft in nude mice, where it grew locally and displayed the same immunophenotype as the original tumor.

Five-week-old athymic swiss mice (Charles River Laboratories, Inc) were housed in the single pathogen free zone of the Animal Facility Laboratories of the Institut de Recerca de Vall d’Hebron and they were provided with food and water *ad libitum*. Prostate tumors were sliced into 5×5 mm pieces and grafted subcutaneously as previously described [33]. The surgical interventions were performed under isofluorane anesthesia and meloxicam analgesia, and all efforts were made to minimize suffering. The protocol was approved by the CEEA (Comitè Étic Experimentació Animal) of the Research Institute Hospital Vall d’Hebron (Permit number 14/05 CEEA). All experimental procedures were conducted in accordance with institutional standards, which fulfilled the requirements established by the Spanish Government and the European Community (Real Decreto 223/1988 and BOE 256, 10/25/90). Tumor growth was assessed by measuring two perpendicular diameters with a caliper. The volume (V) of each tumor was measured once a week and was calculated as previously described [33]. The median of tumor volumes was calculated for each group of mice.

### Animal Treatment

Mice were divided into four groups: a) mice injected with 6 mg of streptozotocin (STZ) (Sigma-Aldrich, Madrid, Spain) intraperitoneally (IP), one week before the tumor was grafted; b) mice treated with STZ IP after tumor implantation; c) mice treated with the vehicle (citrate buffer 50 mM pH 4,5) IP and d) non-treated mice. Blood samples were taken twice a week by saphenous vein for measurements of their glycemia as a control of the maintenance of diabetic status. At the end of the experiments, treated and control animals were sedated by CO^2^, and immediately sacrificed by cervical dislocation. Tissue were collected and frozen at −80° or in formalin.

### Histology

Tumor samples were fixed with formalin and paraffin-embedded; then five µM-thick tumor sample sections were hydrated with xylene (3×10 min) and ethanol at decreasing concentrations (100%, 90%, 70%, 30%; 2×5 minutes each) and stained with hematoxilin (30 seconds) (Surgipath Medical Industries, Inc., IL, US) and eosin (20 seconds) (Sigma-Aldrich). Finally, samples were dehydrated with graded ethanol solutions (as described above) and cleared in xylene before being mounted with DPX (Panreac SA, Barcelona, Spain).

### Immunohistochemistry

After the de-waxed and hydration processes, the slides were pre-treated using a microwave antigen retrieval method (Dako Diagnostics SA, Barcelona, Spain). Endogenous peroxidase was quenched before overnight incubation at 4°C with anti androgen receptor (rabbit polyclonal AR C-19, Santa Cruz Biotechnology Inc., Heidelberg, Germany) used at 1∶50 and with monoclonal mouse anti-human cytokeratin, (clones AE1/AE3; Dako, Denmark). After several washes, sections were incubated 30 minutes at room temperature with secondary antibodies conjugated with HRP (Dako Diagnostics SA), and reactions were developed for 1 min with diaminobenzidine and the H_2_O_2_ system. Appropriate negative controls were performed incubating sections without the primary antibody.

### RNA Analysis

Total RNA was extracted from LNCaP D-glucose and L-glucose treated cells using RNeasy Mini kit, (QIAGEN SL, Madrid, Spain) Reverse transcription (RT) was performed at 42°C, for 50 min using 2 µg of total RNA and 200 U of Superscript II together with an oligo-dT primer and reagents provided by Invitrogen. An aliquot of the RT product was amplified in a 25- µl reaction using SYBRGreen (Invitrogen SA, Barcelona, Spain) with appropriate oligonucleotide primer pairs corresponding to human androgen receptor (forward primer 5′-TGAAAGCCATGCTACTCTTCAG-3′ and reverse primer 5′-GCTCA CCATGTGTGACTTGAT-3′), human 18S (forward primer 5′- TAACGAACG AGACTCTGGCAT-3′ and reverse primer 5′-CGGACATCTAAGGGCATCACAG-3′). Results were analyzed using the 7000 SDS program and quantified by the comparative C_T_ method (2^−ΔΔC^_T_ method).

### Western Blot Analysis

After treatments, LNCaP cells were washed twice with cold PBS, scraped off the flask and homogenized in RIPA buffer supplemented with Complete™ protease inhibitor cocktail (Roche Diagnostics, Barcelona, Spain). Protein extracts were used for Western blotting with antibodies against AR rabbit polyclonal antibody AR N-20 (Santa Cruz Biotechnology Inc), human phospho-NF-κB (sc-33039; Santa Cruz Biotechnology Inc.) and PPIA rabbit polyclonal antibody (SA-296; BIOMOL Int., Madrid, Spain) at 4°C. After several washes, immunoreactive bands were visualized by horseradish peroxidase-conjugated secondary antibody (Dako Diagnostics SA) followed by peroxidase-dependent chemiluminescent reagent (Pierce Biotechnology Inc., Barcelona, Spain) by exposure to x-ray film. Densitometric analysis was performed using ImageJ program.

### Statistical Analysis

The results are expressed as mean ± SD. Data were subjected to the ANOVA test and significance (p<0.05; p<0.01) was attained.

## Results

### Insulin Treatment has no Effect on Androgen Receptor (AR) Levels in LNCaP Cells

**Figure 1:**
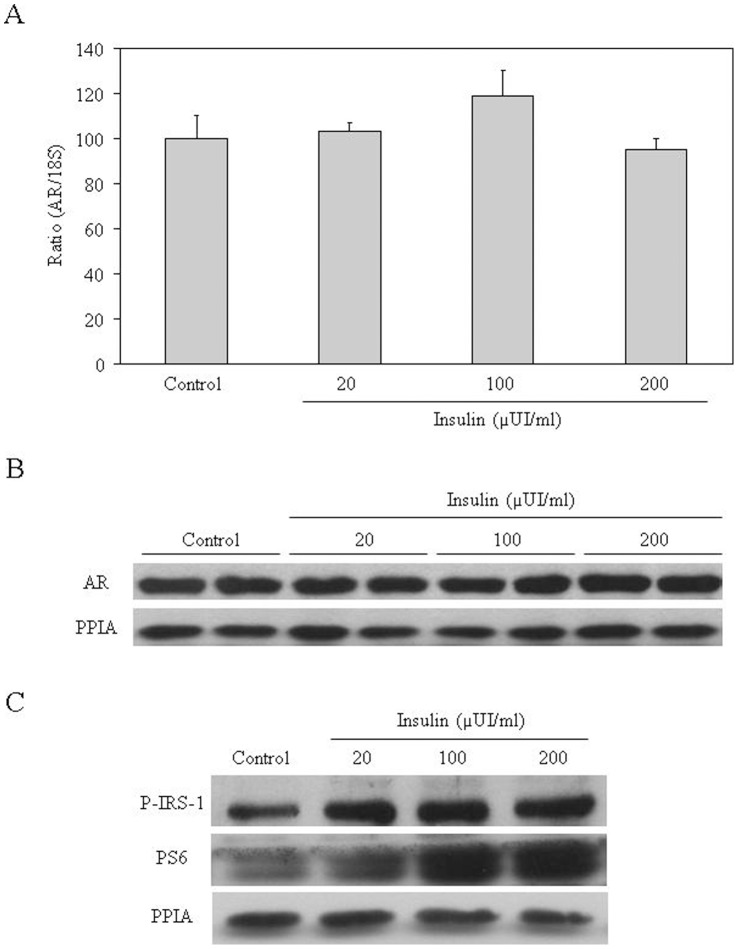
Hyperinsulinemia does not change androgen receptor levels in LNCaP cells. (**A**) Relative AR mRNA levels in the presence of different insulin concentrations are shown. Human 18 S mRNA was amplified as a control. Data points are shown as mean ± SD of triplicates. (**B**) Analysis of AR protein levels in the presence of different insulin concentrations in LNCaP cells by western blot. PPIA has been used as a housekeeping reference protein. (**C**) Phosphorylation levels of IRS-1 and PS6 in LNCaP cells treated during 4 days with different concentrations of insulin measured by western blotting. PPIA has been used as a housekeeping reference protein.

Since PCa is androgen dependent, we first wanted to examine if insulin had any effect on AR levels. We treated LNCaP cells with different insulin concentrations (20, 100 or 200 µUI/ml) for four days. The results showed that insulin treatments did not change AR mRNA levels when compared with untreated LNCaP cells (Figure 1A). Moreover, AR protein levels were also unchanged by insulin treatment when compared with controls (Figure 1B). The effectiveness of insulin treatment was determined by measuring the phosphorylation of IRS-1 and PS6 proteins. The results showed that insulin treatment increased phosphorylation of both proteins when compared to the control LNCaP cells (Figure 1C).

### Increasing Glucose Concentrations Downregulate AR in LNCaP Cells

**Figure 2:**
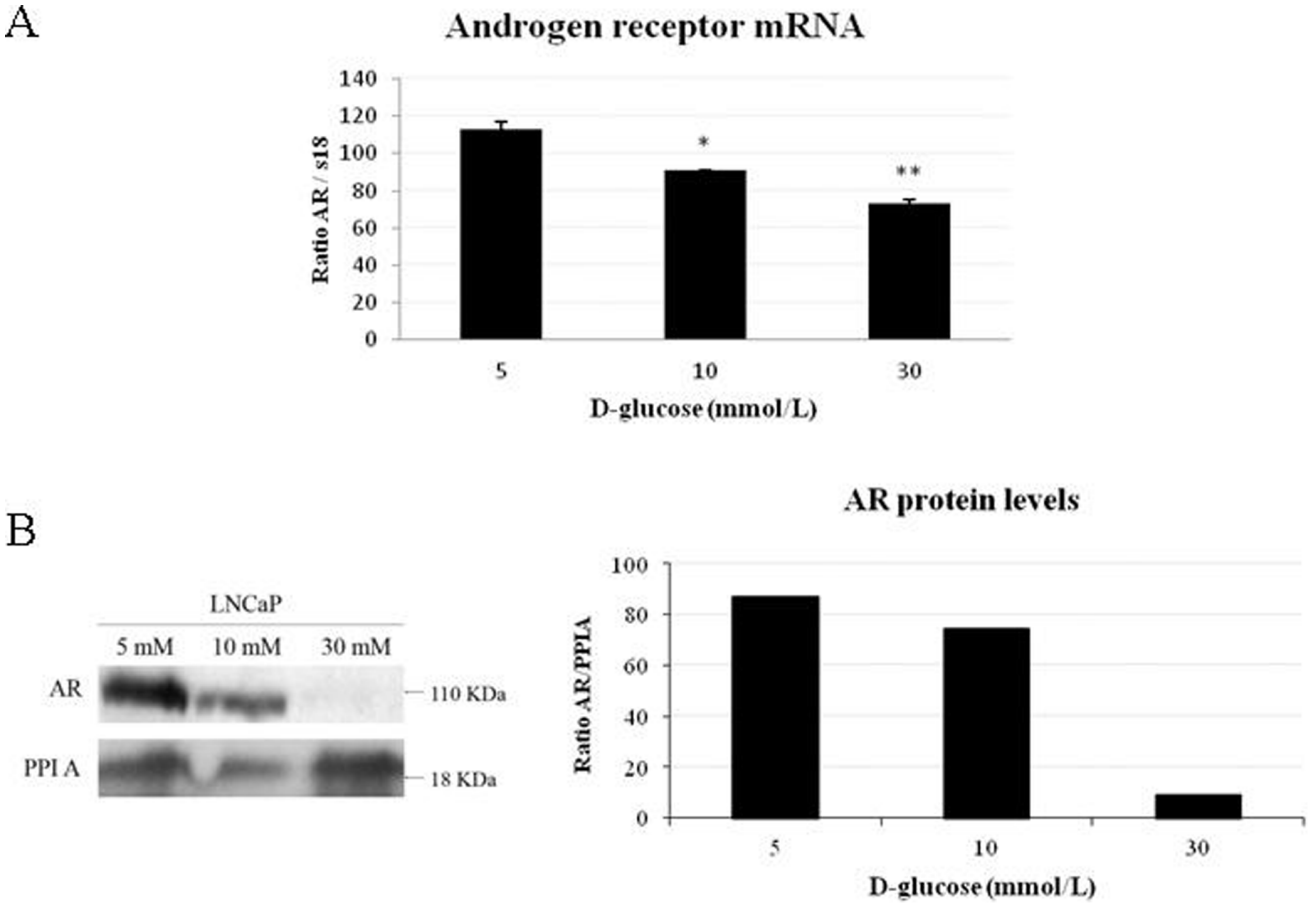
Hyperglycemia downregulates androgen receptor levels in LNCaP cells. (**A**) Relative AR mRNA levels in the presence of different D-glucose concentrations are shown. Human 18S mRNA was amplified as a control. Data points are shown as mean ± SD of triplicates. (**B**) Analysis of AR protein levels in the presence of different D-glucose concentrations in LNCaP cells by western blot. PPIA has been used as a housekeeping reference protein.

We next wanted to study the effects of hyperglycemia on AR levels using LNCaP cells. LNCaP cells were cultured with increasing concentrations of glucose (5 mM, 10 mM and 30 mM) over the course of four days. The media was changed everyday to maintain constant glucose concentrations. AR mRNA levels were reduced significantly in LNCaP cells cultured in 10 or 30 mM glucose when compared to LNCaP cells cultured in 5 mM glucose (Figure 2A). Moreover, AR protein levels were also significantly reduced in LNCaP cells cultured in 10 or 30 mM glucose when compared with LNCaP cells cultured in 5 mM glucose (Figure 2B). To rule out any osmotic effect of glucose, we repeated the experiments using L-glucose and we did not find any decrease in AR protein levels (data not shown).

### The Synergic Effect of High Glucose Concentrations and TNFα in the Downregulation of the AR in LNCaP Cells

**Figure 3:**
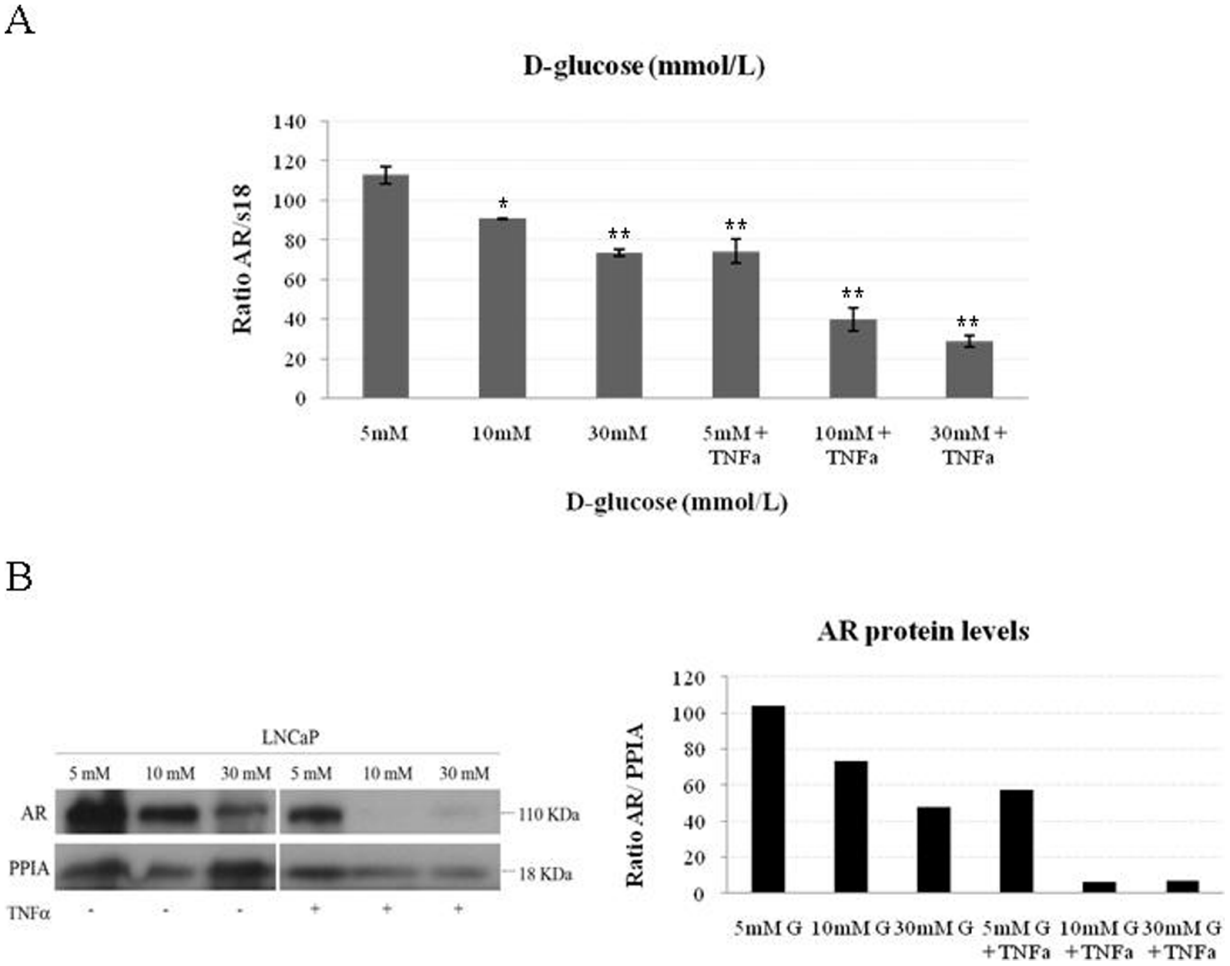
Synergic effects of hyperglycemia and TNFα on the androgen receptor downregulation in LNCaP cells. (**A**) Relative AR mRNA levels in LNCaP treated with an increasing concentration of D-glucose with or without TNFα. Human 18 S mRNA was amplified as a control. Data points are shown as mean ± SD of triplicates. *P<0.05 and **P<0.01 compared with 5 mM of D-glucose. (**B**) Analysis of AR protein levels in LNCaP cells treated as in **A** measured by Western blotting using PPIA as a housekeeping reference protein.

Diabetic patients are characterized by a certain state of low grade inflammation and present high levels of circulating TNF-α in comparison with control subjects. Therefore, we wanted to study the effect of the addition of TNF-α in our *in vitro* system. For this purpose we repeated the glucose treatments in the presence or absence of TNF-α (50 ng/ml). The results showed again that AR mRNA and protein levels were reduced significantly when cells were cultured in 10 or 30 mM glucose when compared with LNCaP cells cultured in 5 mM glucose. Notably, a potent synergic effect leading to a significant further reduction of both AR mRNA and protein levels was obtained when TNFα was added to different D-glucose concentrations in LNCaP cells (Figure 3A and B).

### Hyperglycemia Downregulates AR through NF-κB Activation in LNCaP Cells

To examine whether hyperglycemia was able to downregulate AR through NF-κB activation in our *in vitro* system we performed the following experiments.

**Figure 4:**
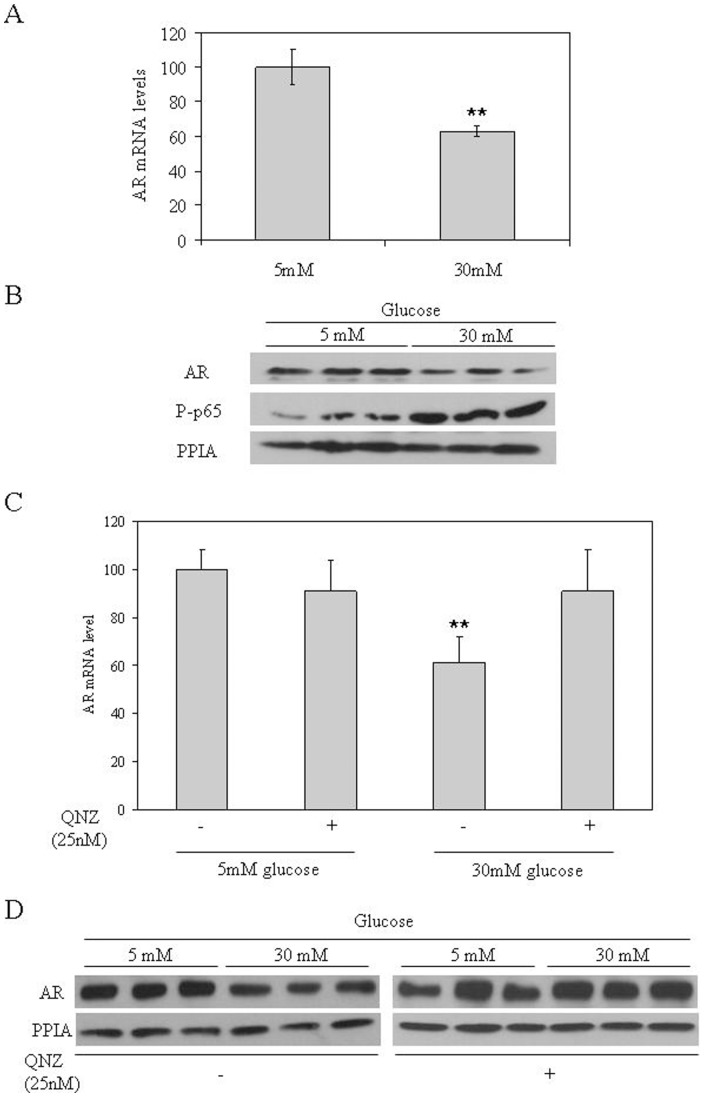
Hyperglycemia reduces androgen receptor levels via NF-κB activation in LNCaP cells. (**A**) Relative AR mRNA levels in LNCaP treated with 5 mM or 30 mM glucose. Human 18 S mRNA was amplified as a control. Data points are shown as mean ± SD of triplicates. *P<0.05 and **P<0.01 compared with 5 mM glucose. (**B**) Analysis of AR and phospho-p-65 protein levels in LNCaP cells treated as in **A** measured by Western blotting using PPIA as a housekeeping reference protein. (**C**) Relative AR mRNA levels in LNCaP treated with 5 mM or 30 mM glucose in the absence or presence of QNZ (25 nM). Human 18 S mRNA was amplified as a control. Data points are shown as mean ± SD of triplicates. *P<0.05 and **P<0.01 compared with 5 mM glucose. (**D**) Analysis of AR protein levels in LNCaP cells treated as in **C** measured by Western blotting using PPIA as a housekeeping reference protein.

We first treated LNCaP cells with 5 mM and 30 mM glucose and mRNA levels of AR were analyzed at baseline and after 4 days of culture. The results showed that 30 mM glucose was able to reduce AR mRNA levels when compared with LNCaP cells grown in 5 mM glucose (Figure 4A). The AR protein levels were also reduced in LNCaP cells grown in 30 mM glucose when compared with 5 mM glucose. More importantly, an increase in p65 phosphorylation, an NF-κB subunit, was also detected in cells grown in 30 mM glucose when compared with cells grown in 5 mM glucose (Figure 4B).

We next decided to treat LNCaP cells with 5 mM or 30 mM glucose in the presence or absence of QNZ (25 nM), an NF-κB inhibitor. The results showed that QNZ co-treatment was able to block the hyperglycemia-induced downregulation of AR mRNA and protein levels (Figure 4C and D).

### Streptozotocin-induced Diabetes (STZ-DM) Produces Prostate Cancer (PCa) Growth Retardation in a PAC120 Mouse Model

**Figure 5:**
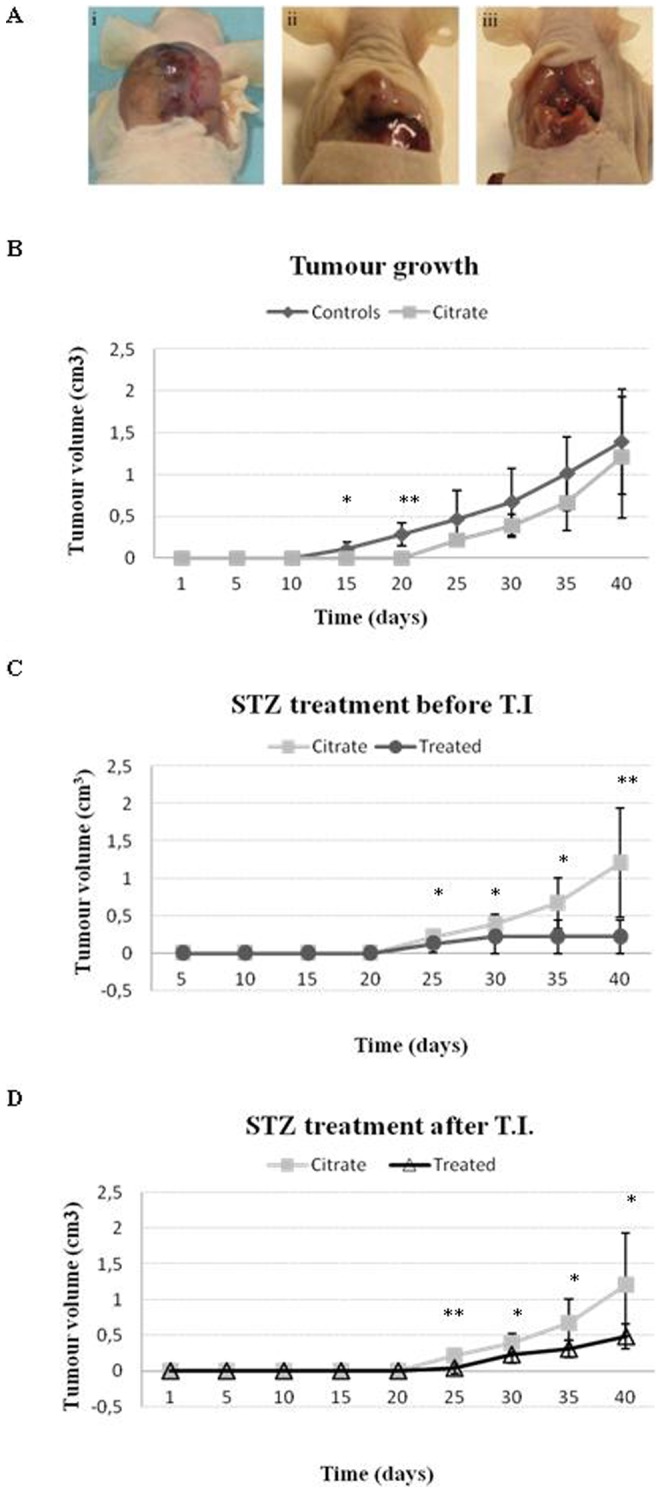
Treatment with streptozotocin reduces prostate tumor growth in PAC120 mouse model. (**A**) Pictures of tumors developed in vehicle treated mice (n = 5) (i), STZ treated after or before tumor implantation (n = 7 for both) with reduced (ii) or no tumor growth (iii). (**B**) Comparison of tumor volume for non-treated (n = 5) and citrate treated (n = 5) mice. (**C**) Tumor volume of STZ-treated animals after tumor implantation compared with tumor volume of citrate treated mice. (**D**) Tumor volume of STZ-treated animals before tumor implantation compared with tumor volume of citrate treated mice. Data are mean ± SD. *P<0.05 and **P<0.01 compared with the control.

We next wanted to study if hyperglycemia by itself could protect against developing prostate cancer *in vivo*. In order to do this, we used a PAC120 PCa mouse model with STZ-DM. For this purpose intraperitoneal injection of STZ was administered to swiss nude mice before (n = 7) and after (n = 7) subcutaneously PAC120 tumor implantation. Non-treated (n = 5) and citrate treated (n = 5) mice were used as controls. Non-treated and citrate treated mice showed a similar tumor growth (Figure 5Ai) while STZ-DM mice showed reduced tumor growth (Figure 5Aii) or no tumor growth (Figure 5Aiii).

Tumor growth was followed by measuring tumor volumes once a week. Non-treated and citrate treated mice showed similar growth during the 40-day experiment (Figure 5B). Both groups of diabetic mice showed reduced tumor growth when compared with the control mice (Figure 5C and D). Mice that were diabetic before tumor implantation (Figure 5D) showed a higher reduction in tumor growth when compared with mice that were diabetic after tumor implantation (Figure 5C).

### Streptozotocin-induced Diabetes (STZ-DM) Reduces AR Expression in Tumors and Endogenous Prostates of PAC120 Mouse Model

**Figure 6:**
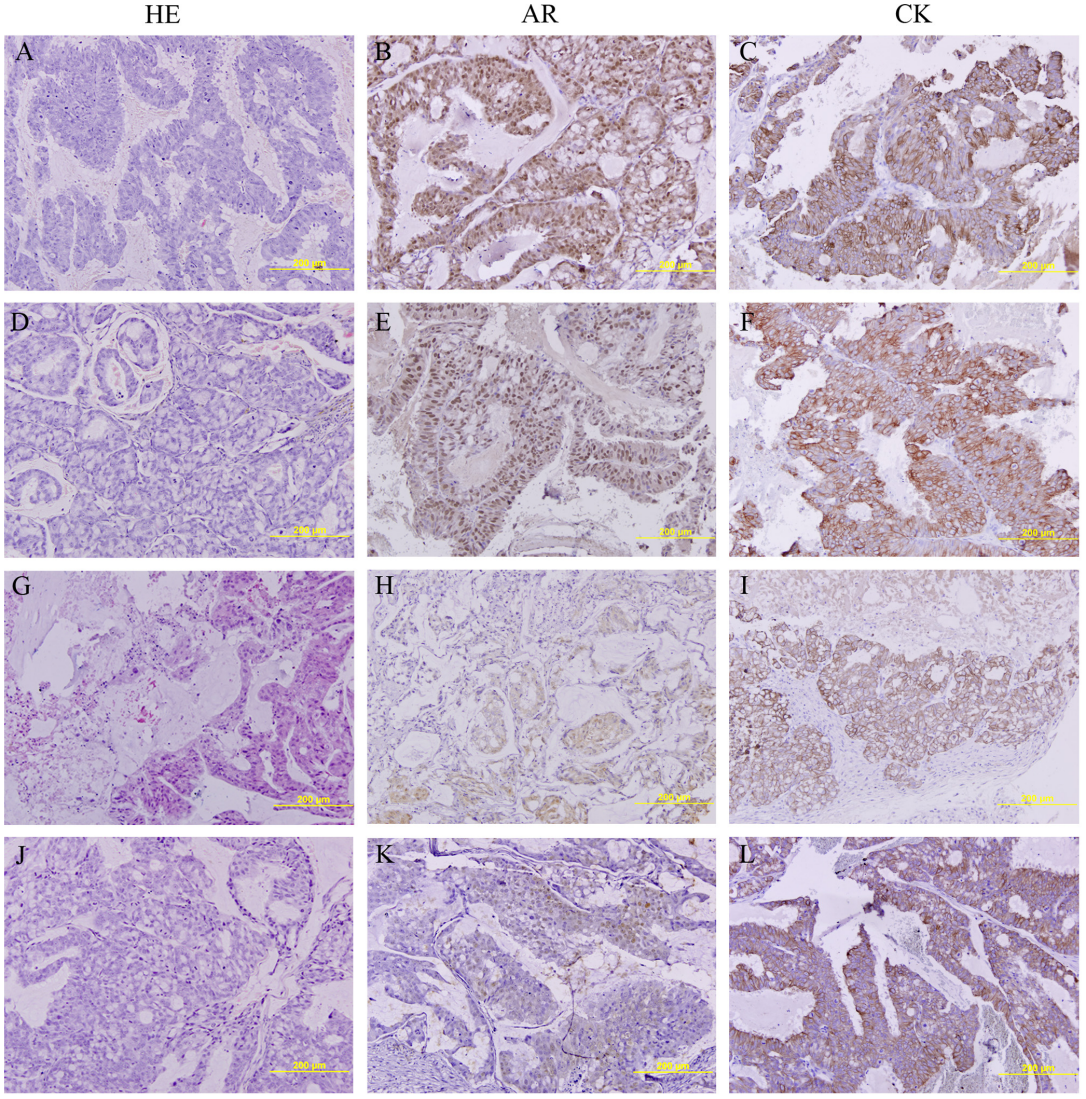
Treatment with streptozotocin reduces androgen receptor staining in prostate tumors in PAC120 mouse model. (**A**) HE staining of prostate tumor xenografts from control mice, (**D**) citrate treated mice, (**G**) STZ-treated before tumor implantation and (**J**) STZ-treated after tumor implantation. (**B**) AR staining of prostate tumor xenografts from non-treated mice, (**E**) citrate treated mice, (**H**) STZ-treated mice before tumor implantation and (**K**) STZ-treated mice after tumor implantation. (**C**) Cytokeratine staining of prostate tumor xenografts from control mice, (**F**) citrate treated mice, (**I**) STZ-treated mice before tumor implantation and (**L**) STZ-treated mice after tumor implantation.

Histological examination of hematoxylin/eosin (HE) stained sections from both groups of diabetic mice (Figure 6G and J) did not show any morphological changes in the tumor in respect to the controls or citrate treated mice (Figure 6A and D). They showed rare glandular differentiation, high mitotic index and were classified as Gleason 9 (4+5) by the pathologist.

Since our *in vitro* experiments showed that hyperglycemia reduced AR levels we decided to explore the possibility that STZ-DM was affecting AR levels *in vivo*. We performed immunohistochemistry against the androgen receptor, in the tumors of control and diabetic mice. Control animals showed positive and strong nuclear staining for androgen receptor in all epithelial cells (Figure 6B and E). Diabetic mice showed epithelial cells with no staining or few cells with nuclear or cytoplasmic AR staining, but the majority of the cells were AR negative (Figure 6H and K). We confirmed that negative AR cells were epithelial by performing an immunohistochemistry against pan-cytokeratin in the tumors of control, citrate and STZ-DM mice (Figure 6C, F, I and L).

**Figure 7:**
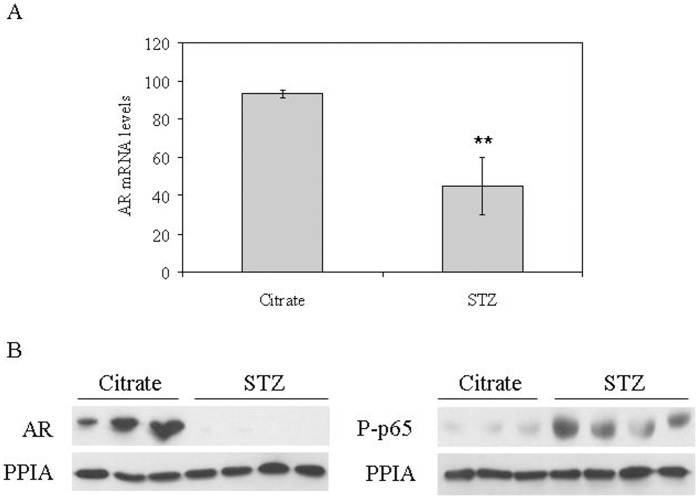
Analysis of the androgen receptor and p65 levels in tumor xenografts of control and STZ treated mice. (**A**) Relative AR mRNA levels for citrate and STZ-treated mice. Human 18 S mRNA was amplified as a control. Data points are shown as mean ± SD of triplicates. (**B**) Analysis of AR (left panel) and phospho-p-65 (right panel) protein levels from citrate and STZ-treated mice measured by Western blotting using PPIA as a housekeeping reference protein.

We next measured AR mRNA levels in xenografts from control and STZ-DM mice and we found that hyperglycemia reduces AR mRNA levels in tumors of STZ-DM mice when compared with control mice (Figure 7A). In addition, as occurs in LNCaP cells a downregulation of AR levels was accompanied by an increase of p65 phosphorylation, an NF-κB subunit, in xenografts from STZ-DM mice (Figure7B).

**Figure 8:**
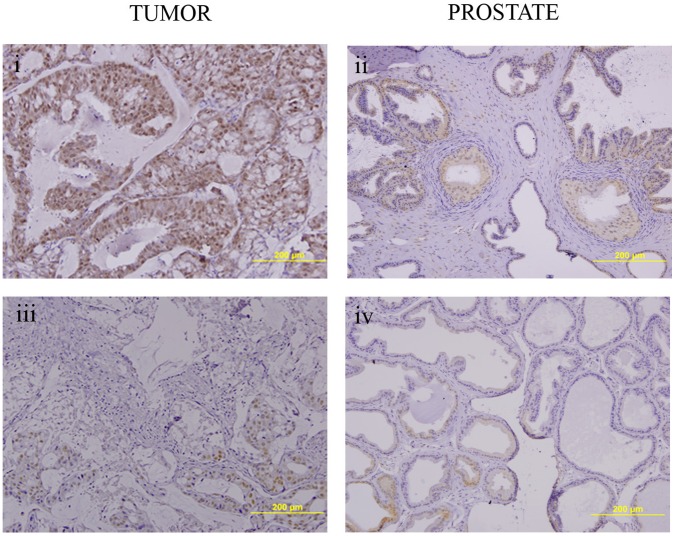
Analysis of the androgen receptor levels in tumor xenografts and endogenous prostates of mice. AR staining is shown in xenograft prostate tumors (i) and in endogenous prostates (ii) from control mice. AR staining is shown in xenograft prostate tumors (iii) and endogenous prostates (iv) from STZ- treated mice.

Finally, we stained the endogenous prostates of control and both groups of diabetic mice to explore whether hyperglycemia also downregulates endogenous AR levels. Indeed, AR staining was reduced in prostates of the STZ-DM mice when compared with the control mice (Figure 8).

## Discussion

PCa is the most common solid organ cancer in men in the USA, Canada and Australia, and the second most common cancer in men globally. US men have a current estimated lifetime risk of one in six and PCa represents, after lung cancer, the second leading cause of cancer-related mortality [1]. Established risk factors of PCa are older age, African-American ethnicity and a history of the disease in a first-degree relative. T2D has been associated with a low risk of developing PCa and this has been recently confirmed in a meta-analysis which included 29 cohort and 16 case-control studies involving 8.1 million participants and 132,331 PCa cases [34]. Our findings give the first experimental support to this concept.

The AR pathway plays a key role in the structural and functional integrity of the prostate, as well as for PCa growth and progression [15]. In this regard, it has been reported that the knockdown of AR leads to growth inhibition and apoptosis of both androgen-dependent and androgen-independent PCa cells [35]–[37]. However, the effect of diabetes on AR and tumor growth had never been explored. In the present study we provide first evidence that hyperglycemia downregulates AR through NF-κB activation, and that this inhibitory effect is further increased by TNFα. In addition, we found that this downregulation of AR induced by diabetes is associated with significant tumor growth retardation in an *in vivo* model of PCa. We would like to point out that in the *in vitro* experiments we have always obtained consistent results in terms of glucose-induced reduction of AR levels. However, we have detected a significant variability in the extent of the reduction of the AR levels caused by the glucose treatment.

Genetic susceptibility and low levels of insulin, IGF-1 and testosterone are among the proposed factors conferring protection for PCa in T2D. By contrast, the possible role of two essential events that occur in T2D: hyperglycemia and the increase of proinflammatory cytokines (ie. TNFα) have not been previously examined.

PCa has one of the strongest relationships between age for any human cancer, and genetic factors are estimated to account for 42% of the risk [15]. It is possible that the presence of genetic factors such as TCF2 variants, which are associated with both T2D and the low risk of PCa participate in the low risk of PCa observed T2D [20]. However, the temporal relationship between DM diagnosis and PCa risk (as the time since DM-diagnosis increases, the risk of PCa decreases) strongly argues against this concept [4]. In support of this we found a lower growth of PCa xenofraft in the group of mice with longer diabetes duration.

Insulin is a growth factor for prostatic epithelium and it also stimulates the growth of a rat PCa cell line in vitro [38]. More recently, an experimental study reported intracellular *de novo* steroidogenesis promoted by insulin in PCa [39] but the effect on AR was not analyzed. These results suggest that insulin may directly promote the proliferation of prostate cancer cells. However, these observations are based on an experimental model for castration-resistant prostate cancer, and the effect of insulin on prostate tumorigenesis during the early phase or in hormone-naïve cancer (such as LNCaP) remains to be elucidated. In addition, the doses of insulin used were supraphysiological and further studies using doses of insulin in the picomolar range (such as was used in the present study) are needed. In the clinical setting, while some prospective studies demonstrate that there may be an association between hyperinsulinemia and PCa risk, there are also several studies that do not support a role for insulin in PCa risk and thus it continues to be an area of investigation [4], [15]. Nevertheless, insulin depletion occurring with increasing duration of diabetes may limit insulin action and hence protect against PCa. In this regard, men with low insulin levels due to diabetes seem to have a decreased risk of PCa development [40], [41]. In fact, the low levels of insulin could be a contributing factor involved in the lower tumor growth observed in PAC120 PCa mice treated with STZ used in the present study.

A proposed hypothesis for how hypoinsulinemia may decrease prostate carcinogenesis is by limiting the bioavailability of insulin-like growth factor I (IGF-I) [15]. However, recent studies suggest that IGF-1 levels are not significantly different between diabetics and controls [41], [42]. In addition, a recent large prospective study has concluded the absence of correlation between the plasma IGF-1 level and insulin resistance [42]. Therefore, the IGF-1-PCa association in type 2 diabetic population remains speculative.

Since PCa is androgen-dependent it is possible that the lower levels of testosterone reported in long-term diabetes (especially obese men) may reach levels below a threshold to a lower risk of PCa [4], [15]. However, it is well recognized that relating a serum testosterone level to a clinical phenotype is an over-simplification, given that circulating testosterone levels, whether free or total, are unlikely to accurately reflect androgen action at the tissue level. Nevertheless, some evidence suggests that low circulating testosterone levels may be a risk factor for PCa aggressiveness [43], [44] and this might be one reason why when PCa appears in T2D it has a worse outcome [15], [44]. In this regard, it is possible that the low AR levels due to hyperglycemia, high TNFα levels and low testosterone levels, apart from conferring protection against PCa development could also act as a mechanism for driving the prostate cells towards some degree of androgen independence. In fact, NF-κB activation has been involved in the androgen-independent growth of PCa [45]. Once a PCa develops in type 2 diabetic patients this would explain its aggressiveness and poor outcome [9]–[11]. To some extent this could also happen in obese patients, since obesity may reduce the risk of non-aggressive PCa, while at same time promoting the risk of aggressive PCa [46], [47].

The mechanisms underlying hyperglycemia on AR regulation have not been previously reported. In the present study we found that hyperglycemia downregulates AR through NF-κB activation, and that this inhibitory effect is further increased by TNFα. In addition, the downregulation of AR induced by diabetes was associated with a tumor growth reduction or even no tumor growth in the PAC120 PCa mouse model treated with STZ. Furthermore, a significant reduction in AR expression in the endogenous prostates of this *in vivo* model was detected. Overall, our results suggest that two essential components of T2D: hyperglycemia and the proinflammatory cytokine TNFα participates in reducing the risk of PCa in T2DM. It should be noted that an increased level of TNFα is a constituent of the low-grade inflammatory process characteristic of T2DM (especially in obese patients) and might be an essential factor accounting for the lower risk of PCa in type 2 in comparison with type 1 diabetic patients.

TNF-α is produced primarily by immune cells but it is also produced by numerous other cell types, including epithelial cells of human prostate cancer [48]. TNF-α binds to the TNF-α-receptor (TNFR1), leading to phosphorylation, ubiquitination and proteasome-mediated degradation of the inhibitor of κB (IκB), which binds to and inhibits nuclear factor-κB (NF-κB) activation by forming a complex in the cytoplasm [25], [49]. Degradation of IκB results in the release of NF-κB, which then translocates to the nucleus. In the nucleus, NF-κB regulates the transcription of target genes, which promote cell proliferation or inflammatory responses. In most cells, TNF-α activates caspase-8 and induces apoptosis only when NF-κB activation is hampered [50]. However, in LNCaP cells, even when NF-κB signaling is activated, TNF-α can still induce cell death in a dose-dependent manner [51]. This apparent discrepancy may be explained by the negative regulation of AR expression by NF-κB [25], [31]. It is worth mentioning that by using QNZ (an NF-κB inhibitor) the hyperglycemia-induced downregulation of AR was blocked, thus underlining the central role of NF-κB in preventing the development of PCa in T2D. However, NF-κB overexpression has been observed in several cohort studies of PCa tissues, including bone metastasis [52]–[55] and it predicts poor outcome in patients with hormone–naïve PCa with high nuclear AR. Therefore, further studies addressed to investigating other potential signalling pathways induced by hyperglycemia resulting in AR downregulation independently of NF-κB activation are needed.

Two further considerations should be made regarding the potential preventive role of TNFα in PCa development in T2D. First, TNFα downregulates SHBG [22], [23] and, therefore, TNFα could have a direct effect by downregulating AR via NF-κB activation and an indirect effect by reducing serum levels of testosterone. Second, TNFα can inhibit Leydig cell steroidogenesis [56] and stimulate aromatase activity [57], the enzyme responsible for the conversion of testosterone to estradiol, thus contributing to the low levels of testosterone observed in T2D.

In conclusion, our results suggest that hyperglycemia and TNF-α play an important role in protecting type 2 diabetic patients against PCa by reducing AR levels via NF-κB activation. Further studies addressed to examining whether other mechanisms of NF-κB activation existing in diabetes such as advanced glycated end products (AGEs) or oxidative stress are involved in the lower risk of PCa observed in T2D are warranted.
